# Experimental data from compartment fires with gas burner and upholstered furniture fuels

**DOI:** 10.1016/j.dib.2023.108934

**Published:** 2023-01-24

**Authors:** Joseph Willi, Daniel Madrzykowski, Mark McKinnon, Nicholas Dow

**Affiliations:** UL Fire Safety Research Institute, 6200 Old Dobbin Lane, Suite # 150, MD 21045, Columbia

**Keywords:** Fire model validation, Compartment fires, Fire investigation, Repeatability, Gas burner, Upholstered furniture

## Abstract

There exists a variety of specialized fire dynamics routines, zone fire models, and field fire models. Many of these heuristics and correlations rely on experimental data from fires fueled by gas burners or liquid pool fires and have had minimal, if any, validation against data from fires with solid, more complex fuels, such as upholstered furniture. One hundred and twenty fire experiments were conducted inside a compartment that contained a single ventilation opening in the form of a doorway that was either open or closed for the entirety of each experiment. The fires were fueled by natural gas burners and upholstered furniture items. The compartment was instrumented throughout with thermocouples, oxygen sampling probes, heat flux gauges (total and radiative), pressure transducers, and bi-directional probes. Additionally, heat release rate data were collected during open door experiments with fires larger than 100 kW. This experimental series was designed to better quantify the repeatability of and differences between natural gas burner and upholstered furniture fuels and to provide new validation cases for the fire modeling community.

**Specifications Table**

**Table:** 

Subject	Engineering
Specific subject area	Experimental Thermal and Fluid Sciences and Fire Safety Engineering
Type of data	Table
How the data were acquired	Data were acquired using type K thermocouples, Servomex O${}_{2}$ Analyzers, Schmidt-Boelter heat flux gauges, Setra Model 264 differential pressure sensors, bi-directional probes, and oxygen consumption calorimetry. The data were recorded using a data acquisition system composed of National Instruments SCXI hardware.
Data format	Raw
Description of data collection	One hundred and twenty fire experiments were conducted inside a 3.7 m by 3.7 m by 2.4 m compartment with a 0.9 m by 2.0 m doorway that was either open or closed for the entirety of each experiment. Gas burners and upholstered furniture placed at different positions in the compartment served as fuels. Replicate experiments were conducted for most fuel, fuel position, and ventilation configuration combinations. Gas temperature, oxygen concentration, heat flux, pressure, and gas velocity were measured at various locations throughout the compartment. Heat release rate data were obtained via oxygen consumption calorimetry during the majority of open door experiments with fires larger than 100 kW.
Data source location	Experiments were conducted at the UL Calorimetry Laboratory in Northbrook, Illinois, United States.
Data accessibility	Raw data are archived in a GitHub repository Data identification number: https://doi.org/10.5281/zenodo.5703475 Direct URL to the data: https://github.com/ulfsri/fsri-compartments-2018 The dataset citation is in Ref [1]

**Value of the Data**

• These data provide a comprehensive view of fluid flow and gas-phase heat transfer in compartment fires with well-defined fuel sources (natural gas burners) and more complex fuel sources (upholstered furniture) at different locations under different ventilation conditions. The data also offer insight into the repeatability of compartment fires fueled by natural gas burners and upholstered furniture.
• These data can benefit fire model developers and practitioners, fire protection engineers, and fire investigators.
• These data can be used to validate predictive fire algorithms and models and better understand their applicability to fires fueled by upholstered furniture. They can also be used to evaluate the repeatability of fire conditions generated by gas burner and upholstered furniture fuels.

## 1 Data Description

The GitHub repository [1] contains the data and supporting files from the 120 compartment fire experiments. The repository contains directories for the raw data and event times, supporting information about the experiments, and a Python script to generate time history charts of the data.

### 1.1 Data

The data directory (01_Data/) contains two comma-separated value (CSV) plaintext files for each experiment. The name of the file containing the time series measurement data from each experiment matches the name of the experiment. The second file for each experiment includes the experiment name followed by _Events and contains the timing information of notable events from the experiment. The name of each experiment includes four parts separated by underscores that correspond to a different aspect of the experimental configuration. The first part of the experiment name identifies the fuel utilized during the experiment. Table 1 lists the abbreviations used for the different fuel sources.

**Table 1:** Abbreviations of the Different Fuels used in Experiment Names.

Abbreviation	Fuel
100kWSB	0.3 m burner set to 100 kW
100kW	0.6 m burner set to 100 kW
250kW	0.6 m burner set to 250 kW
500kW	0.6 m burner set to 500 kW
RedAccent	Red Accent Chair
Overstuffed	Overstuffed Sofa

The second part of the experiment name highlights the status of the compartment door during the experiment (either open or closed). Over the course of the experimental series, fuels were placed at four different locations inside the compartment. These positions—corner, back, side, and center—are referenced in the third portion of each experiment name. To evaluate the repeatability of fire conditions produced by the different fuels, multiple experiments were conducted for most configurations. The final part of the experiment name corresponds to the replicate number of the given configuration (e.g., _1, _2, or _3 for replicate 1, 2, or 3).

### 1.2 Information

The information directory (02_Info/) includes a CSV file (channel_list.csv) that details the location, type of data, and group associated with each sensor. This file is used by the Python script that constructs time history charts of the measurement data to properly map data channels to their respective sensor group, add labels to the chart legends, and assign file names to the generated charts. Also included in this directory is a CSV file (furniture_mass.csv) that includes the initial mass of the furniture for applicable experiments.

### 1.3 Scripts

The scripts directory (03_Scripts/) contains a single Python script that, when executed, generates time history charts of sensor data from each experiment. The script creates a charts directory (04_Charts/) on the same level as the scripts directory. Directories corresponding to the different experiments are created within the charts directory and then populated with portable document format (PDF) files that contain the experimental data plots from the various sensor groups.

## 2 Experimental Design, Materials and Methods

One hundred and twenty fire experiments were conducted inside a compartment with a single doorway. Natural gas burners and two types of furnishings were used as fuel sources and were placed at four different locations throughout the experimental series. Various types of instrumentation were installed throughout the compartment to quantify the fire environment during the experiments.

### 2.1 Structure

**Fig. 1:**
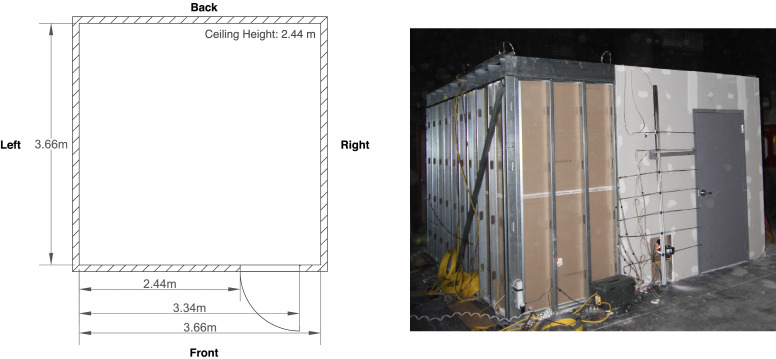
Dimensioned floor plan and image of the compartment utilized during the experiments. The image is a view of the front left corner from the compartment exterior.

All experiments occurred inside a compartment with interior dimensions of 3.7 m long by 3.7 m wide by 2.4 m high. The wall frames of the compartment were constructed from 18 gauge steel studs with an 89 mm web depth that were spaced 41 cm on center. The ceiling frame was constructed from 18 gauge steel joists with a 152 mm web depth that were spaced 41 cm on center. A layer of 16 mm thick Type X gypsum board was secured to the interior sides of the frames. Additionally, the ceiling and walls were lined on the interior with 13 mm thick Type I marinite board, and the floor of the compartment was covered with 13 mm thick Durock cement board. A single ventilation opening was present at the front of the compartment in the form of a doorway measuring 2.0 m high by 0.9 m wide. The door and frame at the opening were composed of steel and had a 45 minute fire rating. The interior side of the door and frame were covered with an approximately 25 mm thick layer of Kaowool. A dimensioned floor plan view and image of the compartment are presented in Fig. 1.

### 2.2 Fuels

**Fig. 2:**
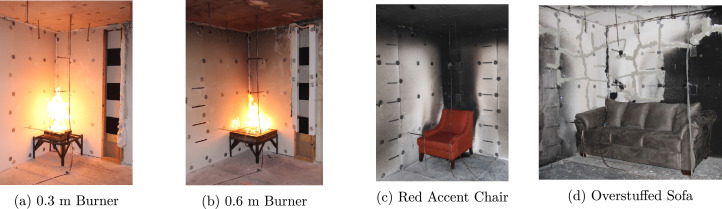
Images of the fuel loads utilized during experiments. Each image shows the fuel load in the corner position.

**Fig. 3:**
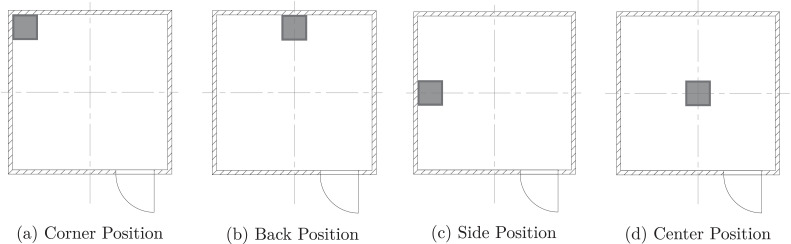
Schematics showing the four positions (represented by the gray squares) of the fuel loads.

The natural gas burners and upholstered furniture pictured in Fig. 2 were used as fuel sources during the experiments. These fuels were positioned at the four locations displayed in Fig. 3 over the course of the experimental series.

Each natural gas burner used as a fuel source was 15 cm tall and had a square opening on its top face. The size of the square opening differed between the two; the side length of the smaller burner’s orifice was 0.3 m, while that of the larger burner was 0.6 m. The burners were supported by a steel frame that elevated them so that their top surface was 0.5 m above the floor. The side length of the square openings is used to differentiate between the two burners—“0.3 m Burner” refers to the smaller burner and “0.6 m Burner” refers to the larger burner. The flow rate of natural gas to the burner was controlled by an Alicat MCR-5000 SLPM mass flow controller. The burners were calibrated under a ventilation hood instrumented with an oxygen consumption calorimeter, and it was determined that the flow rates required to generate 100 kW, 250 kW, and 500 kW fires were 180 SLPM, 420 SLPM, and 843 SLPM, respectively.

**Fig. 4:**
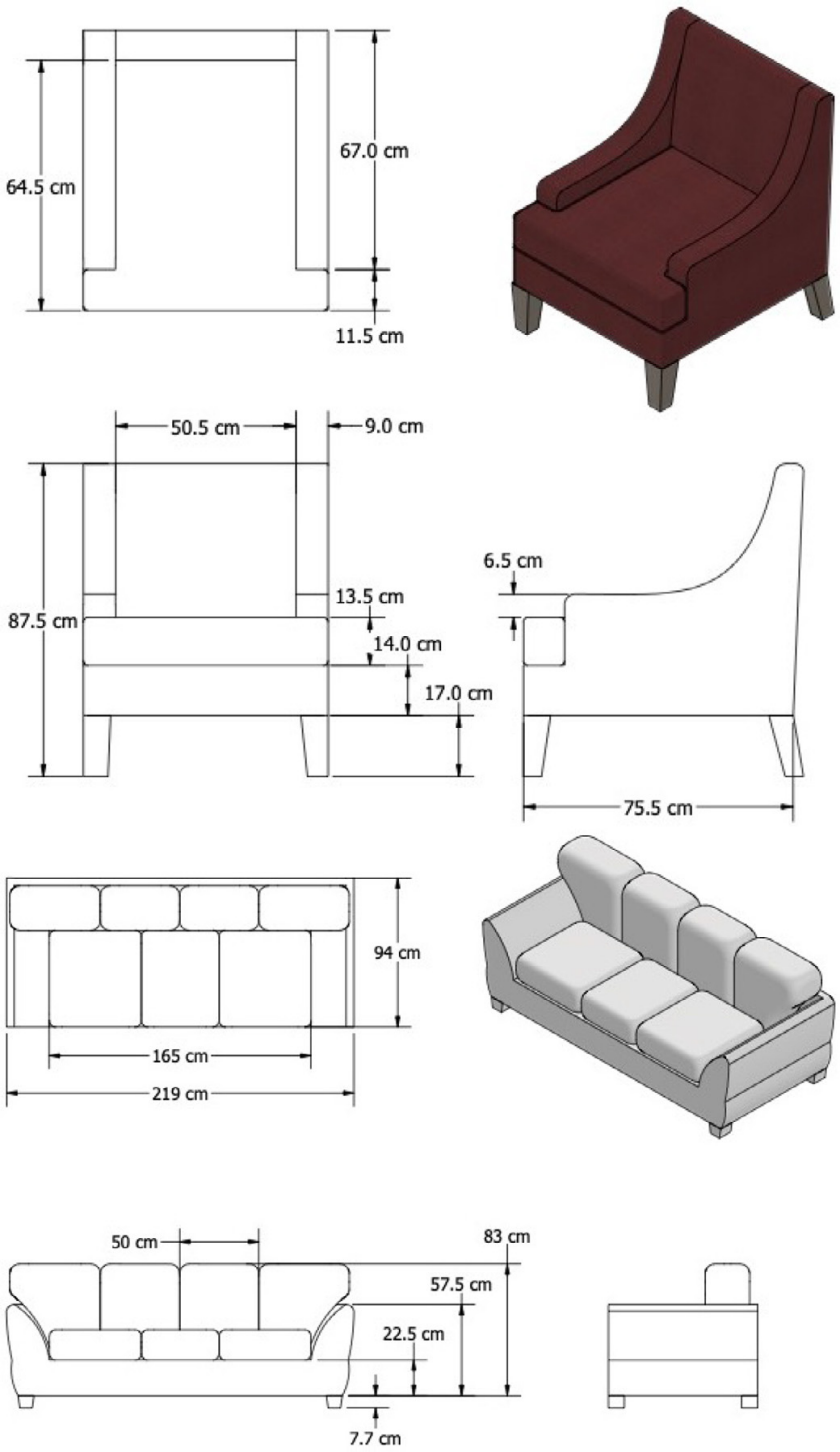
Dimensioned drawings of the Red Accent Chair and Overstuffed Sofa.

In addition to the gas burners, an upholstered chair and an upholstered sofa, referred to as “Red Accent Chair” and “Overstuffed Sofa”, were used as fuel loads during experiments. Both furniture items had a polyester (PE) outer covering, a wooden frame, back and arm cushions comprised of PE batting, and seat cushions composed of polyurethane foam covered by PE batting on the top and bottom. The average mass of the chair and sofa were 20.4 kg ± 0.3 kg and 49.1 kg ± 0.8 kg, respectively. Dimensioned drawings of the two furniture items are presented in Fig. 4.

**Fig. 5:**
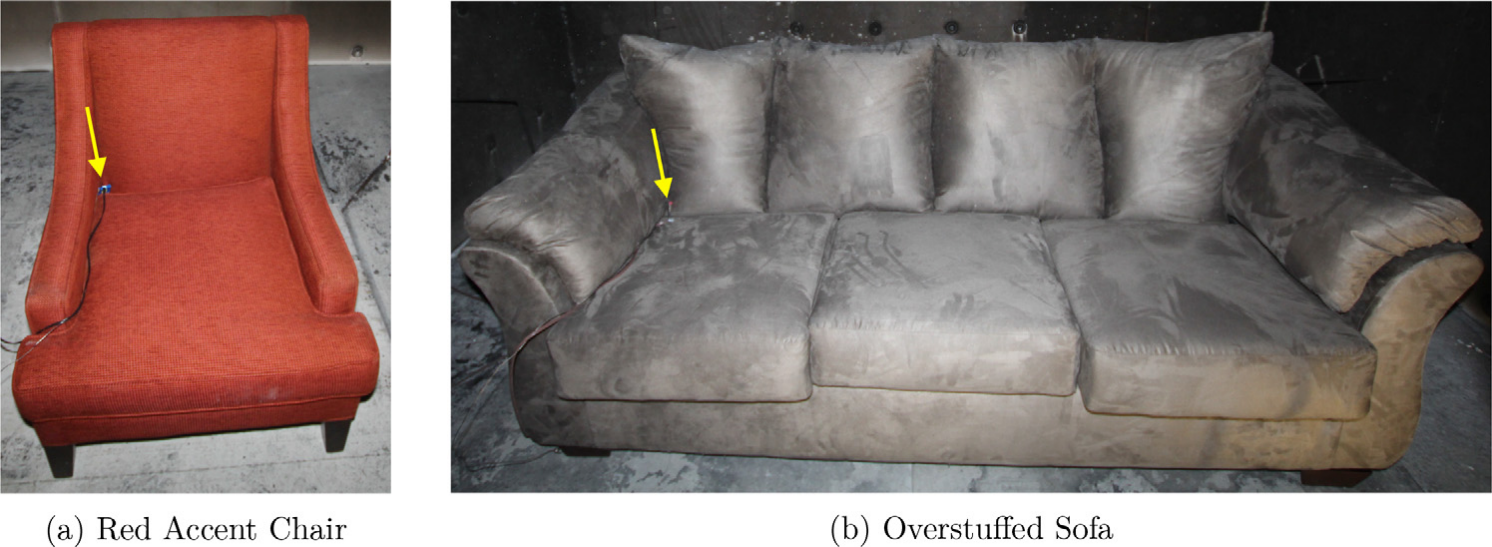
Ignition locations for the furniture experiments.

The furnishings were always oriented so that their backside was facing the back wall of the compartment. Ignition occurred via an electronic matchbook placed on the seat cushion against the back and arm cushions. For all experiments with the Red Accent Chair, the matchbook was placed at the location highlighted in Fig. 5a. For experiments with the Overstuffed Sofa at the corner and center locations, the matchbook was placed at the position shown in Fig. 5b. During experiments at the back location, the sofa was in the same physical position as the corner location (i.e., against the left and back walls). However, the matchbook was positioned at the opposite end of the sofa so that the point of ignition was aligned with the back fuel load position.

### 2.3 Instrumentation

**Fig. 6:**
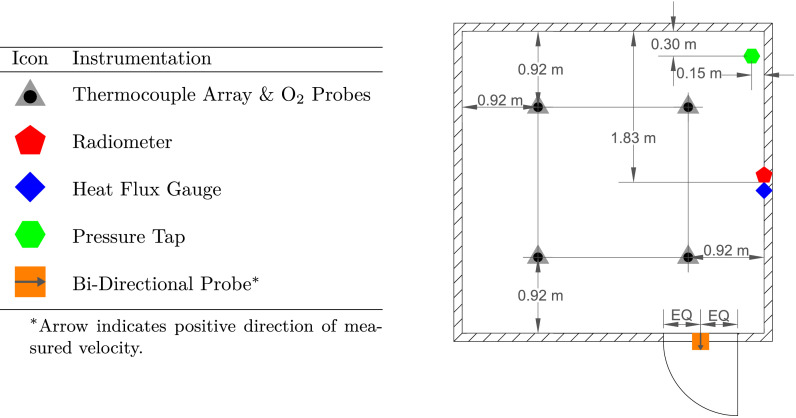
Floor plan showing the locations of the fixed instrumentation throughout the compartment.

**Fig. 7:**
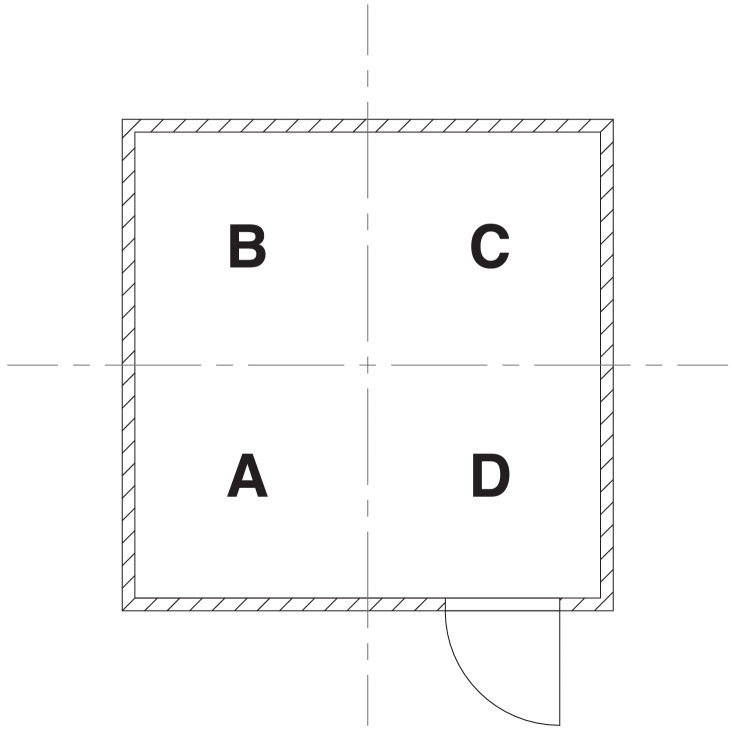
Floor plan showing the quadrants within the compartment.

Different types of instrumentation were installed throughout the compartment to measure gas temperature, oxygen concentration, heat flux (total and radiative), pressure, and gas velocity. A floor plan showing the locations of the fixed instrumentation within the compartment is presented in Fig. 6. During experiments where the fuel was against a wall (i.e., at the corner, back, or side location), additional heat flux gauges and bi-directional probes (BDPs) were positioned to measure the total heat flux to the adjacent wall along with the gas velocity and temperature within the fire plume. Names were assigned to sensors and their respective groups based on dividing the compartment into the quadrants presented in Fig. 7.

A vertical array of type K, bare-bead thermocouples with 0.5 mm nominal diameters was installed in the center of each quadrant. The arrays are represented by gray triangles in Fig. 6. Each array contained a total of eight thermocouples that were positioned 25 mm below the ceiling and at 0.3 m increments below the ceiling. The total expanded uncertainty of the thermocouple measurements is estimated to be ± 15 % based on previous studies from Blevins and Pitts et al. [2], [3]. Additionally, gas samples were collected through pairs of stainless steel tubes co-located with these thermocouple arrays (black circles in Fig. 6) and positioned 0.6 m and 1.8 m below the ceiling. After passing through the steel probes, the gas samples were drawn through a coarse, 2 micron paper filter followed by a condensing trap to remove moisture. Then, they passed through a high-efficiency particulate air filter before the oxygen concentration was measured by a Servomex O${}_{2}$ Analyzer. During experiments with the furniture fuels, the sampling probes 1.8 m below the ceiling in quadrants B and D were repositioned to be 2.1 m below the ceiling and 33 cm from the left and right walls, respectively. Based on a study by Lock et al., the estimated total expanded uncertainty of the O${}_{2}$ concentration data is ± 12 % [4].

Water-cooled Schmidt-Boelter gauges (nominal diameter of 25 mm) were utilized to measure the radiative and total (convective and radiative) heat flux to the compartment walls. Zirconium plates were installed over the faces of some Schmidt-Boelter gauges to prevent contributions from convective heat transfer and ultimately transform the gauges into radiometers. Total and radiative heat flux to the right side wall were measured by gauges centered along the wall and positioned 0.65 m and 1.3 m below the ceiling. These total heat flux gauges and radiometers, represented by the blue diamond and red pentagon in Fig. 6, were installed so that their faces were flush with the interior side of the wall. Additionally, a pair of gauges at identical heights were used to measure the total heat flux from the fire plume during experiments in which the fuel load was against a wall (i.e., at the corner, back, or side location). These two gauges were flush with the interior side of the wall adjacent to the fuel source and were aligned with the centerline of the fire plume. Results from an international study on total heat flux gauge calibration and response demonstrated that the total expanded measurement uncertainty of a Schmidt-Boelter gauge is typically ± 8 % [5].

Setra Model 264 differential pressure transducers (measurement range of ± 125 Pa) connected to copper sampling probes via polyvinyl tubing were used to measure the pressure inside the compartment during the experiments. Three sampling probes positioned 0.3 m, 1.2 m, and 2.1 m below the ceiling were installed at the location marked by the green hexagon in Fig. 6. The total expanded uncertainty of the pressure measurements is estimated to be ± 10 % [6].

Seven BDPs paired with type K, inconel-sheathed thermocouples with nominal diameters of 1.6 mm were utilized to measure gas velocity through the compartment doorway during open door experiments. These BDPs, represented by the orange square in Fig. 6, were installed along the exterior side of the compartment. They were horizontally-centered in the doorway and spaced 25 cm apart between the doorway lintel and the floor. The stainless steel probes were connected to Setra Model 264 differential pressure transducers that were identical to those previously mentioned. Additionally, during experiments in which the fuel load was against a wall (i.e., at the corner, back, or side location), two BDPs paired with thermocouples located 0.65 m and 1.3 m below the ceiling were positioned over each fuel load to measure the gas velocity and temperature within the fire plume. A previous gas velocity measurement study focused on flow through doorways during pre-flashover compartment fires yielded total expanded uncertainties ranging from ± 14 % to ± 22 % for measurements from BDPs similar to those described here [7]. Therefore, the total expanded uncertainty for gas velocity measured during these experiments is estimated to be ± 18 %.

Lastly, the heat release rate was measured via oxygen consumption calorimetry during the majority of open door experiments with fires larger than 100 kW. The laboratory calorimeter was designed for a maximum fire size of 10 MW. The smoke collection exhaust hood had a diameter of 7.6 m, was positioned approximately 7.6 m above the floor, and operated at 14 m${}^{3}$/s to ensure capture of the combustion products during the experiments. In a previous study, Bryant and Mullholland estimated the total expanded uncertainty of oxygen consumption calorimeter data from full-scale fire experiments to be ± 11 % [8]. The study considered numerous sources of uncertainty for the analysis and identified those associated with the generic value for heat produced per mass of oxygen consumed and with determining the mass flow rate of exhaust gases as the main contributors to the total expanded uncertainty estimate.

A National Instruments data acquisition system was used to record all instrumentation data. The system consisted of a SCXI-1001 chassis, a SCXI-1600 control module, and SCXI-1102C 32-channel voltage input modules (± 10 V) connected to TC-2095 rack mount adapters that provided built-in cold junction compensation for thermocouples. Data were sampled at 1 Hz for all experiments.

### 2.4 Procedure

As mentioned in Section 2.2, the fuels were placed at four locations inside the compartment over the course of the experiments, and the compartment door was either open or closed for the entirety of each experiment. Three replicates were conducted for the majority of the tested configurations to assess the repeatability of each setup. The experiments are summarized in Table 2, which contains a list of the tested configurations organized by fuel type. For example, consider the first line that summarizes the 24 experiments conducted with the 0.3 m Burner at 100 kW. Eight unique configurations are represented by the “Door Status” and “Fuel Position(s)” columns—four open door configurations (one for each fuel position) and four closed door configurations (one for each fuel position). The “Experiments per Configuration” column indicates three experiments with each of the eight configurations were performed, which results in a total of 24 experiments represented by the one line. Note, the values in the “Total Experiments” column of the table are cumulative.

**Table 2:** Summary of Experiments.

Fuel	Door Status	Fuel Position(s)	Experiments per	Total
			Configuration	Experiments
0.3 m Burner at 100 kW	Open, Closed	Corner, Back, Side, Center	3	24
0.6 m Burner at 100 kW	Open, Closed	Corner, Back, Side, Center	3	48
0.6 m Burner at 250 kW	Open, Closed	Corner, Back, Side, Center	3	72
0.6 m Burner at 500 kW	Open, Closed	Back, Side, Center	3	90
0.6 m Burner at 500 kW	Open, Closed	Corner	1	92
Red Accent Chair	Open, Closed	Corner, Center	3	104
Red Accent Chair	Open, Closed	Back	1	106
Overstuffed Sofa	Open, Closed	Corner	3	112
Overstuffed Sofa	Open	Back	3	115
Overstuffed Sofa	Closed	Back	1	116
Overstuffed Sofa	Open	Center	1	117
Overstuffed Sofa	Closed	Center	3	120

At least two minutes of background data was collected before ignition during each experiment. The method and timing implemented for extinguishment after ignition varied on the experimental configuration. During open door experiments with the gas burners, the fire was extinguished via the stoppage of gas flow after temperatures throughout the compartment were determined to be at a quasi-steady state for at least three minutes. The extinguishment time usually ranged between 10–15 minutes after ignition. For the open door experiments with the furniture fuel loads, the fires were allowed to grow to a fully-developed state and then decay into a fuel-limited state before the smoldering remains were suppressed. Alternatively, the closed door experiments were designed to study ventilation-limited fires. Thus, fires during these experiments were allowed to burn until there were no visible signs of flaming combustion due to oxygen depletion.

## Ethics Statements

This work did not involve human subjects, animal experiments, or data collected from social media platforms.

## CRediT authorship contribution statement

**Joseph Willi:** Formal analysis, Investigation, Resources, Data curation, Writing – original draft, Visualization. **Daniel Madrzykowski:** Conceptualization, Methodology, Resources, Project administration, Funding acquisition. **Mark McKinnon:** Writing – review & editing. **Nicholas Dow:** Investigation, Resources.

## Declaration of Competing Interest

The authors declare that they have no known competing financial interests or personal relationships that could have appeared to influence the work reported in this paper.

## Data Availability

2018 Compartment Fire Experimental Data (Original data) (Zenodo).

## References

[1] J. Willi, D. Madrzykowski, M. McKinnon, N. Dow, 2018 Compartment Fire Experimental Data (v2.0), 2021, 10.5281/zenodo.6376302 PMC9926187 36798602

[2] Blevins L. (1999). Proceedings of the 33^rd^National Heat Transfer Conference.

[3] Pitts W., Braun E., Peacock R., Mitler H., Johnson E., Reneke P., Blevins L. (2003). Proceedings of the 14^th^Joint Panel Meeting.

[4] Lock A., Bundy M., Johnsson E., Hamins A., Ko G., Hwang C., Fuss P., Harris R. (2008). NIST Technical Note.

[5] Pitts W., Murthy A., de Ris J., Filtz J., Nygård K., Smith D., Wetterlund I. (2006). Round robin study of total heat flux gauge calibration at fire laboratories. Fire Saf. J..

[6] Kerber S., Madrzykowski D. (2008). NIST Technical Note.

[7] Bryant R. (2009). A comparison of gas velocity measurements in a full-scale enclosure fire. Fire Saf. J..

[8] Bryant R., Mullholland G. (2008). A guide to characterizing heat release rate measurement uncertainty for full-scale fire tests. Fire Mater..

